# Controlled Atom Transfer Radical Depolymerization

**DOI:** 10.1002/advs.77962

**Published:** 2026-09-27

**Authors:** Dimitra Mantzara, Richard Whitfield, Glen R. Jones, Nghia P. Truong, Athina Anastasaki

**Affiliations:** ^1^ Laboratory of Sustainable Polymers, Department of Materials ETH Zurich Zurich Switzerland

**Keywords:** atom transfer radical polymerization, chemical recycling, depolymerization

## Abstract

Atom transfer radical polymerization is a versatile controlled radical polymerization methodology in which control over polymerization is achieved by shifting the activation‐deactivation equilibrium towards dormant species, leading to polymers of targeted molecular weight and dispersity. However, the chemical recycling of ATRP materials lacks this level of control, with current methodologies favoring rapid end‐group activation over deactivation, resulting in an uncontrolled depolymerization whereby polymer chains unzip completely without any molecular weight control. In this work, the first example of controlled ATRP depolymerization is realized by strategically promoting deactivation over both activation and depropagation. The controlled nature of the reaction is achieved by increasing catalyst concentrations and reducing temperatures, resulting in on‐demand and uniform shortening of the polymer chains during the depolymerization, as evidenced by a linear decrease in molecular weight with conversion. Furthermore, while the uncontrolled depolymerization of diblock copolymers results in the simultaneous release of both monomers, controlled depolymerization leads to selective regeneration of the second block, where the monomer closer to the halogen end‐group is preferentially released first, thus providing a powerful analytical handle for structural characterization. The versatility of this strategy is confirmed across various activation mechanisms, including thermal, photochemical, and initiators for continuous activator regeneration (ICAR) ATRP.

## Graphical abstract

Controlled atom transfer radical depolymerization allows for precise, uniform shortening of polymer chains to yield monomers on demand. By tuning catalyst concentration and temperature, this strategy prioritizes deactivation to maintain molecular weight control throughout depolymerization. Additionally, block copolymers undergo selective, sequential monomer release from the halogen chain‐end, providing an analytical tool for structural characterization.

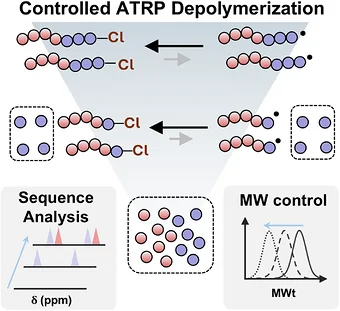

## 1 Introduction

Reversible deactivation radical polymerization (RDRP), often also known as controlled radical polymerization (CRP), is a powerful synthetic platform that has facilitated the production of polymers with precisely defined molecular weights, narrow molar mass distributions, and well‐controlled architectures [1, 2, 3, 4, 5, 6, 7]. The precision of RDRP systems arises from a dynamic equilibrium which is established between active and dormant species, shifted significantly toward the dormant state. In atom transfer radical polymerization (ATRP), one of the most versatile RDRP techniques, the equilibrium is governed by reversible deactivation of propagating chains with halides, mediated by a transition metal catalyst [8]. By promoting deactivation over propagation, the polymer chains grow uniformly (i.e. low dispersity) while the molecular weight increases linearly with conversion, indicating good control over the polymerization. Another advantage of favoring the dormant state is that the radical concentration is kept low, and hence termination is less likely, yielding, nearly exclusively, halide‐capped polymers. This high end‐group fidelity has been extensively utilized for post‐polymerization functionalization, grafting, and the synthesis of well‐defined block copolymers [9, 10, 11, 12].

These same labile halide end‐groups have also recently been leveraged to facilitate efficient chemical recycling of ATRP‐synthesized materials via depolymerization back to monomer [13, 14, 15, 16]. By catalytically reactivating halide chain‐ends under thermodynamically appropriate conditions, depropagation occurs at significantly lower temperatures than conventional pyrolysis, which typically requires temperatures exceeding 400°C to initiate random backbone scission [17, 18, 19]. In ATRP‐type depolymerization, the transition metal catalyst abstracts the terminal halogen to regenerate the depropagating radical, which leads to the rapid and complete unzipping of polymer chains back to monomer at temperatures between 100 and 170°C. Ouchi and co‐workers first demonstrated this approach using chlorine‐capped poly(methyl methacrylate) (PMMA) in the presence of a ruthenium catalyst at 100°C and 10 mM initial polymer concentration, which yielded 24% monomer recovery after 24 h [20]. Subsequent studies by the Matyjaszewski group achieved significantly enhanced yields of 67–76% at 170°C using either copper or iron catalytic systems [13, 21]. Furthermore, ATRP depolymerization has been successfully conducted in bulk, where the equilibrium was driven toward monomer recovery through the continuous removal of the generated monomer [22, 23, 24]. More recent advancements have utilized light, [25] electrochemistry [26], or external radical sources [27] to (re)generate activating species and achieve high depolymerization conversions even at temperatures as low as 100°C.

**FIGURE 1:**
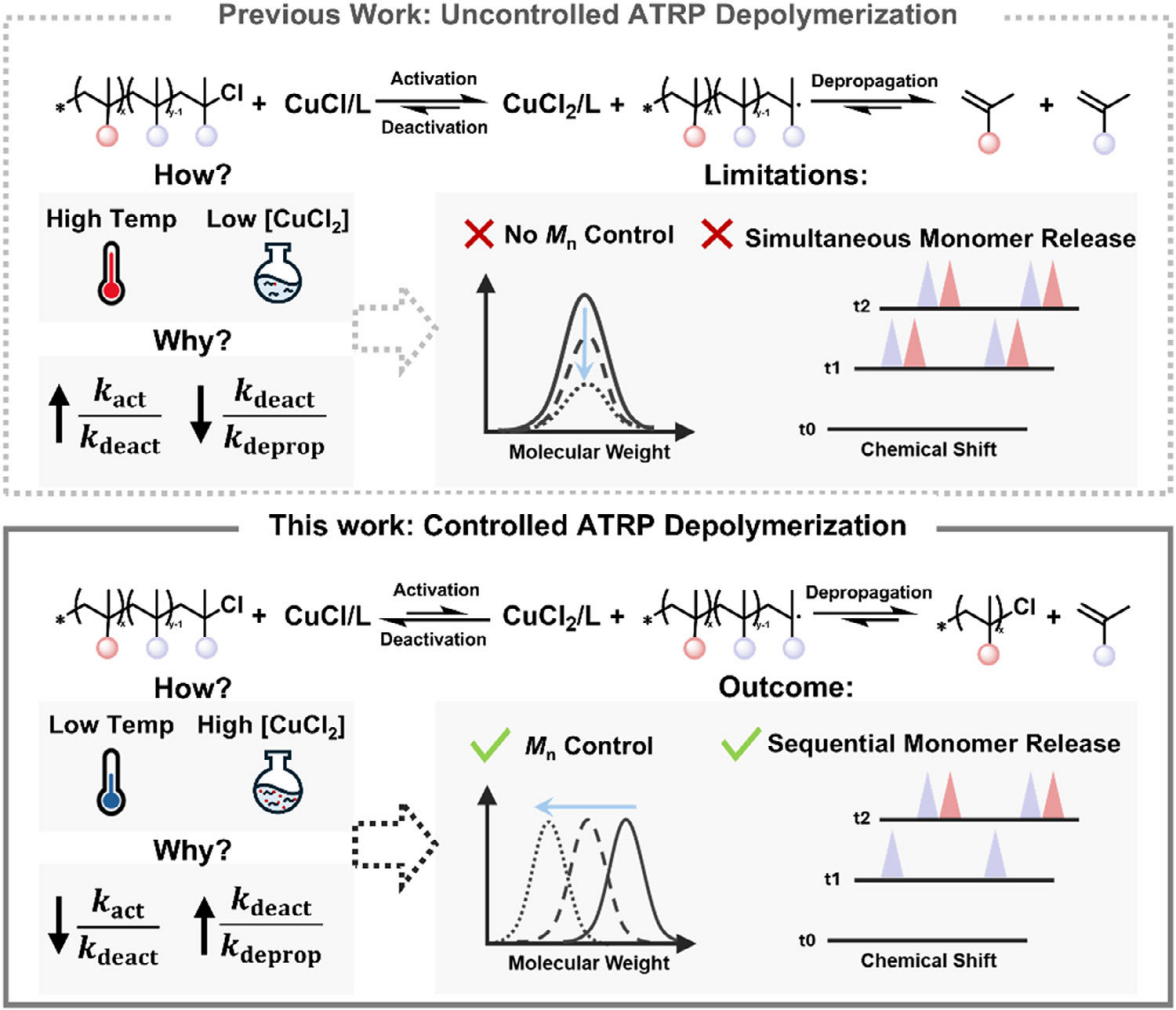
Conceptual figure depicting the differences between uncontrolled (top) and controlled (bottom) ATRP depolymerization.

Despite these advancements, the dynamic equilibrium between active and dormant species in ATRP depolymerization systems is heavily shifted to the active side, thus not exhibiting the control over molecular weight that is characteristic of the forward ATRP mechanism [15]. Monitoring depolymerization reactions via size exclusion chromatography (SEC) typically reveals little to no change in the number‐average molar mass (*M*_n_) throughout the process (Figure 1) [13, 21, 28, 29]. Instead, a steady decrease in peak intensity is observed as the reaction progresses, suggesting that once a polymer chain is activated, it rapidly unzips to monomer without sufficient deactivation to regulate the depropagation step. If ATRP depolymerization were a true reversal of the polymerization process, it would be expected that chains should be reversibly deactivated as they depropagate. Under such conditions, all chains would depropagate concurrently, facilitating a gradual decrease in *M*_n_ relative to depolymerization conversion. Establishing this level of control represents a unique advantage of RDRP‐derived polymers. Unlike the indiscriminate chain scission characteristic of pyrolysis, a controlled depolymerization offers a streamlined methodology to tune molecular weight‐dependent properties on demand. For example, a higher molecular‐weight polymer could be partially depolymerized to a specific lower *M*_n_ to meet target requirements for viscosity, strength, or toughness without necessitating a total resynthesis. Furthermore, controlled depolymerization could provide a precise means for the post‐modification of block copolymers, making it possible to selectively depolymerize one block while leaving the other intact, or to shorten a specific segment to alter properties such as hydrophilicity or self‐assembly characteristics. Although in RAFT depolymerization, control can be induced by simply increasing the initial polymer concentration, in ATRP this “trick” cannot be applied, most likely due to the very high depolymerization rates [30, 31, 32, 33]. Instead, despite the many parameters available to fine‐tune the ATRP equilibrium such as the choice of metal, ligand, or catalyst concentration, control over ATRP depolymerization has never been demonstrated.

Herein, we report the first controlled ATRP depolymerization of polymethacrylates. We demonstrate that by strategically manipulating the ATRP equilibrium through increased catalyst concentrations and reduced reaction temperatures, deactivation of chains can be promoted over activation and depropagation. This shift toward the dormant species facilitates a linear decrease in molecular weight with conversion and represents the first true reversal of the ATRP mechanism. The effectiveness of this controlled system is further highlighted by the depolymerization of an AB‐type diblock copolymer, which exhibits the sequential release of monomers from the chain‐end rather than simultaneous unzipping. Finally, we show that these principles can be universally applied across different ATRP depolymerization systems, as evidenced by similarly high levels of control achieved under thermal, photochemical, and ICAR activation mechanisms.

## 2 Results and Discussion

To distinguish between controlled and uncontrolled depolymerization, we established two primary analytical criteria. First, for homopolymers, a controlled process must exhibit a decrease in *M*_n_ relative to depolymerization conversion. For example, 30% depolymerization conversion would result in a 30% *M*_n_ shift, increasing proportionally to 60% *M*_n_ shift at 60% conversion. This suggests concurrent, uniform unzipping of the entire chain population, whereas an uncontrolled system results in a constant *M*_n_ as individual chains convert to monomer fully upon a single activation event. Second, for AB‐type diblock copolymers, a controlled system would facilitate a sequential release of monomers. In this ideal case, the monomer located at the active *ω*‐end group is released first, with the second monomer appearing only at higher conversions as the depolymerization front reaches the block junction. Consequently, tracking the change in monomer ratio over time provides direct spectroscopic evidence of a controlled depolymerization. In contrast, an uncontrolled process would exhibit simultaneous release of both monomers, where the molar ratio of the recovered monomers should remain relatively constant throughout the depolymerization and reflect the initial block composition of the starting material. To evaluate these criteria, we synthesized two chlorine‐terminated polymers, one homopolymer and one diblock copolymer, to serve as model polymers. Poly(benzyl methacrylate) (PBzMA) was synthesized via activators regenerated by electron transfer (ARGET) ATRP polymerization in anisole at 70°C. The model homopolymer exhibited low dispersity (*Đ* = 1.17) and a high chain‐end fidelity of 90% calculated from chain‐extension (Figures S1‐S3). Additionally, PMMA‐*b*‐PBzMA‐Cl was synthesized by chain‐extending a PMMA‐Cl macroinitiator with benzyl methacrylate (BzMA) under similar ARGET conditions. Size exclusion chromatography (SEC) analysis confirmed a narrow molar mass distribution (*Đ* = 1.11), and ^1^H NMR spectroscopy established a 52/48 (BzMA/MMA) block ratio (Figures S4 and S5, Table S1).

To establish a baseline for the degree of control in a typical ATRP depolymerization, we subjected the Cl‐capped PBzMA homopolymer to standard literature depolymerization conditions. Utilizing the high temperature protocol reported by our and Matyjaszewski's group [14, 21] we achieved 84% conversion within 10 min. The reaction was conducted in trichlorobenzene (TCB) at 170°C with [RU]_0_ = 50 mM in the presence of 0.22 eq. CuCl_2_ and 1.3 eq. tris(2‐pyridylmethyl)amine (TPMA) with respect to the end‐group and was monitored by SEC and ^1^H NMR spectroscopy. The SEC traces showed a minor decrease in molecular weight during the initial stages of depolymerization and a maximum of 20% *M*_n_ shift at 84% conversion, indicating a predominantly uncontrolled system (Figure S6, Table S2). The modest *M*_n_ shift is attributed to a combination of limited deactivation events, and the accumulation of irreversible termination during the high‐temperature depolymerization. To more clearly elucidate the degree of control under these standard conditions, we subsequently monitored the depolymerization of the PMMA‐*b*‐PBzMA‐CI copolymer via ^1^H NMR spectroscopy (Figure S7). The evolution of the BzMA/MMA ratio as a function of time allowed us to confirm the uncontrolled nature of the depolymerization. At the early stages of the reaction (conversion ∼7%), the vinyl peaks of both monomers were observed in the NMR spectra, signifying simultaneous monomer release as expected from an uncontrolled process. However, the initial monomer ratio did show a slight enrichment in BzMA, suggesting that although deactivation events were occurring, their frequency was insufficient to give sole depolymerization of the BzMA block or significantly influence the molar mass distribution of the polymer (Figure S7).

**FIGURE 2:**
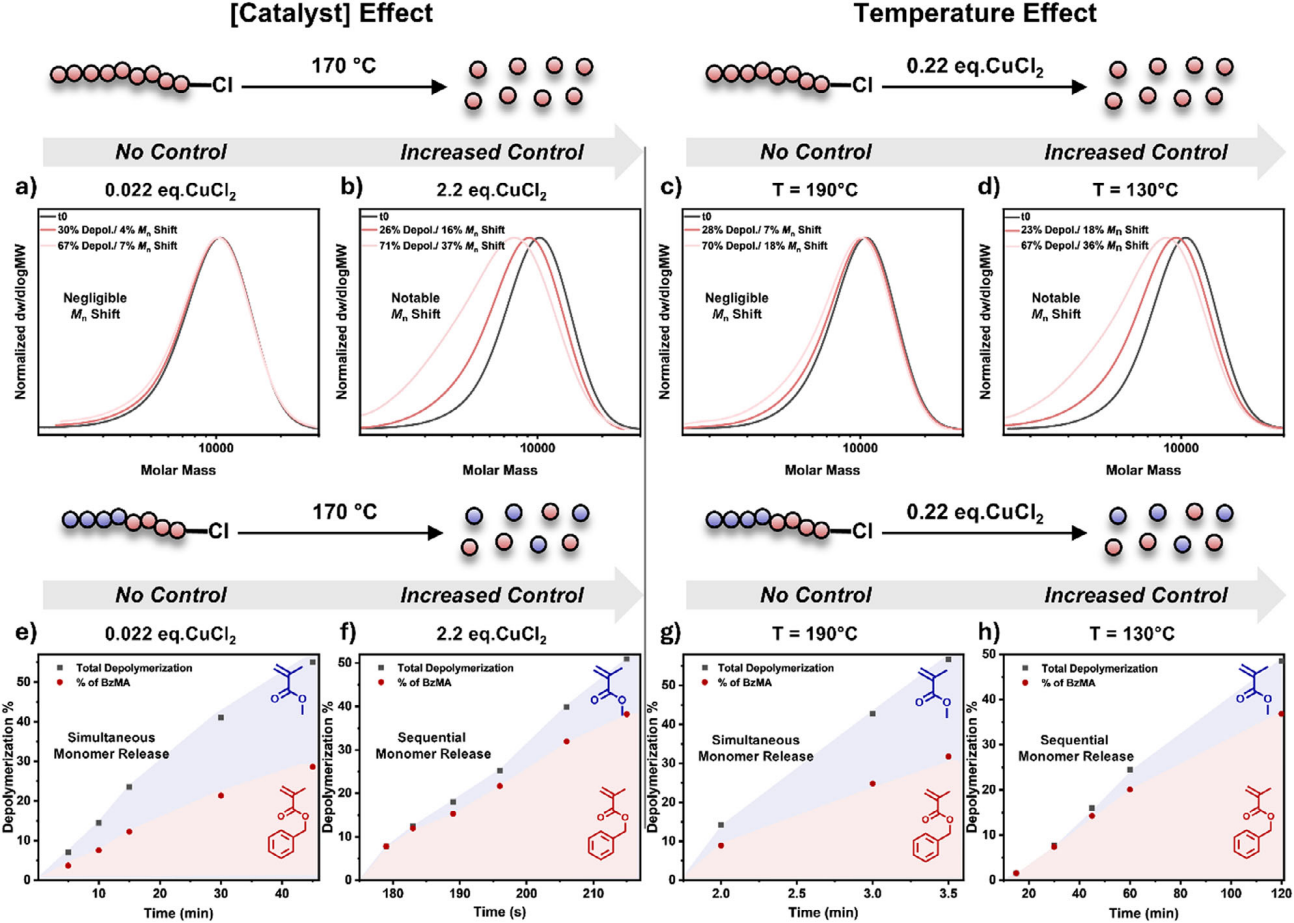
The effect of catalyst concentration (left) and temperature (right) on control during homopolymer (above) and block copolymer (below) depolymerization.

We hypothesized that we could increase the deactivation during depolymerization by increasing the concentration of CuCl_2_/TPMA catalyst, which in typical ATRP reactions serves as the deactivator. The strong dependence of control on the deactivator concentration is well known in ATRP polymerization literature [34]. According to Le Chatelier's principle, by increasing the deactivator concentration, the equilibrium is shifted towards the dormant species. This means that the polymer chains would be rapidly capped upon activation, allowing only a small amount of monomer to be released with each activation cycle, thus facilitating a more uniform chain length reduction. Conversely, decreasing the [CuCl_2_] will shift the equilibrium towards the active depropagating chains, lowering the deactivation rate, and resulting in an uncontrolled reaction where the polymer chains are more likely to rapidly depropagate without mediating deactivation cycles. To explore this hypothesis and investigate the effect of catalyst concentration, we repeated the homopolymer depolymerization, first reducing the CuCl_2_ amount by 10‐fold (0.022 eq. CuCl_2_ with respect to the end group), while maintaining the same conditions ([RU]_0_ = 50 mM, CuCl_2_/TPMA = 1/6, T = 170°C). SEC traces were obtained at various stages of the depolymerization, revealing negligible *M*_n_ reduction (7%), even when the depolymerization had reached high conversion (Figure 2a, Table S3). Additionally, depolymerization of the diblock copolymers exhibited simultaneous monomer release, with the BzMA/MMA ratio remaining stable and close to the initial block ratio (MMA/BzMA = 48/52) throughout the process (Figure 2e, Figure S8). These findings suggest a highly uncontrolled system in which polymer chains are activated and rapidly unzip back to monomer, since the CuCl_2_ amount is not enough to provide sufficient deactivation.

Pleasingly, in agreement with our hypothesis, a more controlled depolymerization reaction was accomplished when higher amounts of CuCl_2_ were employed, under otherwise identical conditions ([RU]_0_ = 50 mM, CuCl_2_/TPMA = 1/6, T = 170°C). Notably, when the CuCl_2_ concentration was increased by 10‐fold (2.2 eq. CuCl_2_, 13 eq. TPMA with respect to end group), the homopolymer depolymerization kinetics revealed a gradual decrease in *M*_n_, with 16% *M*_n_ shift at 26% conversion, which increased to 37% when the conversion reached 71%, as shown in Figure 2b (Table S4). This represents a notable advancement over RAFT depolymerization, which is frequently restricted to lower overall conversions when deactivation is favored [30]. The increased control of the system was confirmed by monitoring the monomer release during depolymerization of the diblock copolymer. During the early stages of the reaction (≤20% conversion), the vast majority of vinyl peaks observed in ^1^H NMR spectroscopy were from BzMA. This promising result suggests the possibility of selective block depolymerization, where the monomer closer to the halogen end‐group is first released exclusively, a clear sign of controlled depolymerization. After this initial regime (<20% conversion), more MMA began to appear, and the BzMA:MMA ratio gradually decreased toward the initial block ratio (52/48) as the reaction progressed (Figure 2f, Figure S9).

Having established that catalyst concentration plays a key role in facilitating controlled depolymerization, we next investigated the effect of temperature on the reaction kinetics and equilibria. It is well established in the polymerization literature that temperature influences the *K*_ATRP _of polymerization reactions [35, 36]. However, existing studies focus on polymerization‐appropriate temperatures, which are typically much lower than those used in ATRP depolymerization. At first, we conducted a series of polymerization experiments under ARGET conditions at temperatures between 70°C and 170°C. We observed that the dispersity of the obtained polymer was higher at elevated temperatures (Figure S10, Table S5), indicating that control is partially lost. This was attributed both to a higher *K*_ATRP_ value and a higher propagation rate, meaning that per activation cycle, more monomer units can be added in a propagating polymer chain, resulting in non‐uniform chain growth. We reasoned that the effect should be similar for the reverse depolymerization reaction, explaining why we do not observe control over depolymerization at 170°C even when increasing the deactivator concentration by 10‐fold. It was also assumed that enhanced control could be achieved when targeting lower temperatures.

To test this hypothesis, we performed depolymerization experiments over a range of temperatures between 190°C and 130°C, keeping other conditions constant across all experiments ([RU]_0_ = 50 mM, 0.22 eq CuCl_2_ / 1.3 eq TPMA, TCB). The magnitude of the *M*_n_ shift became more pronounced as the temperature was lowered, indicating that reduced temperatures are essential for maintaining a controlled depropagation process. For example, major disagreement between *M*_n_ shift and conversion was observed at 190°C, with only 7% *M*_n_ reduction observed at 28% depolymerization, increasing to a maximum of just 18% at 70% conversion (Figure 2c, Table S6). At 130°C, the gap between the theoretical and experimental *M*_n_ shift narrowed significantly, representing a marked improvement in control over the high‐temperature conditions. At 23% conversion, 18% *M*_n_ shift was observed, which increased to 36% *M*_n_ shift at 67% depolymerization (Figure 2d, Table S7). Further lowering the temperature to 120°C also enhanced control (25% *M*_n_ reduction at 45% conversion, Figure S11), but drastically limited the reaction rate, which we attribute to the slow reduction of Cu(II) under these conditions [37]. Applying lower temperature conditions to the AB‐type diblock copolymer confirmed the critical role of temperature in controlling depolymerization. At 190°C, the depolymerization kinetics and the relative BzMA/MMA ratios indicated predominantly simultaneous release of both monomers, suggesting that activation leads to rapid, unregulated unzipping along the entire chain (Figure 2g, Figure S12). In contrast, lowering the temperature to 130°C established a more controlled regime. Under these conditions, at low depolymerization conversions, mostly the BzMA monomer was detectable via ^1^H NMR spectroscopy, with the MMA content slowly increasing as the reaction progressed (Figure 2h, Table S8).

**FIGURE 3:**
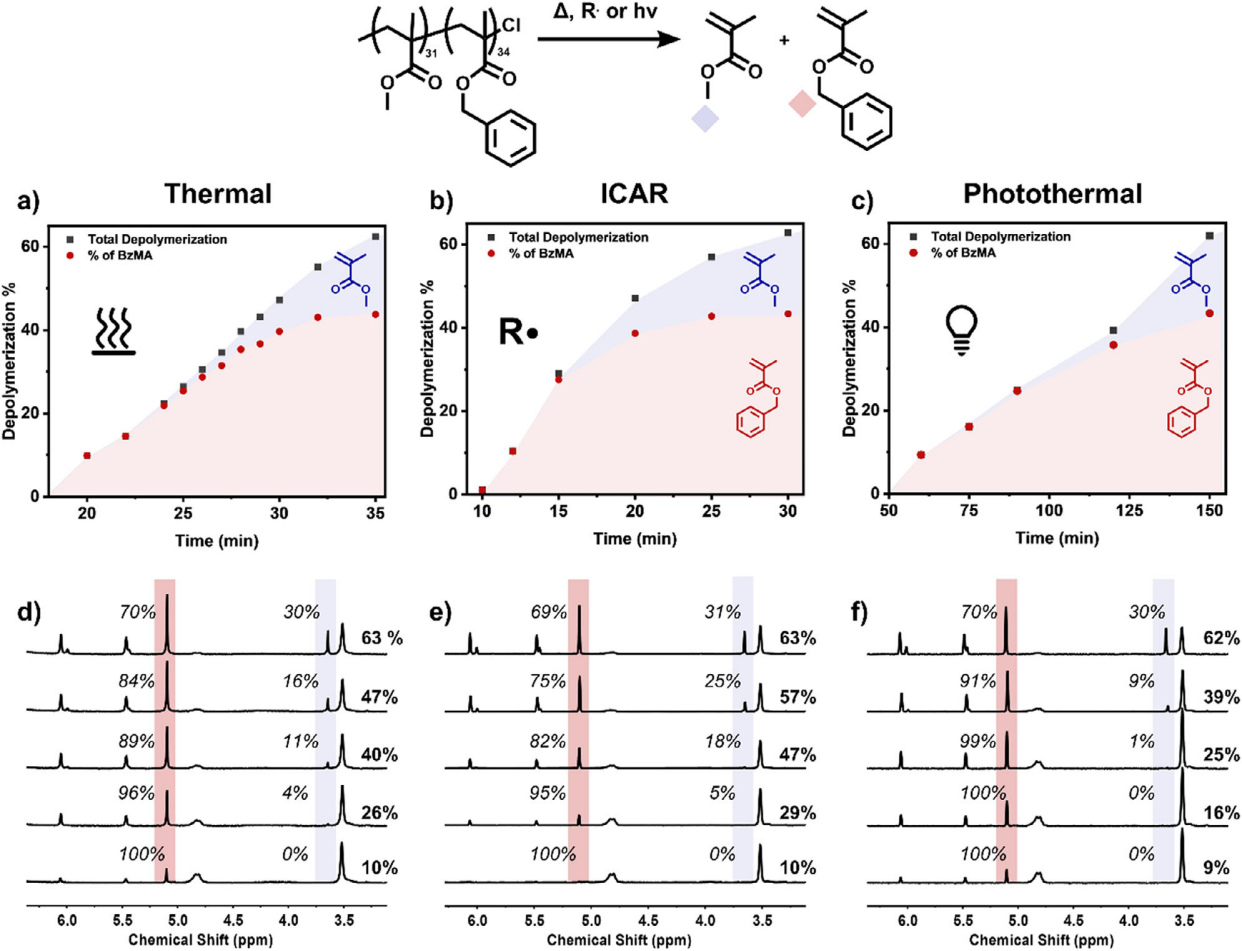
a) Evolution of the monomer release during the thermal ATRP depolymerization of PMMA‐*b*‐PBzMA in TCB at 130°C in the presence of 2.2 eq. CuCl_2_ and an excess of TPMA (13 eq.) b) Evolution of the monomer release during the ICAR – ATRP depolymerization of PMMA‐*b*‐PBzMA in TCB at 120°C in the presence of 2.2 eq. CuCl_2_, 2.2 eq. TPMA and 2.2 eq. ABCN c) Evolution of the monomer release during the photothermal ATRP depolymerization of PMMA‐*b*‐PBzMA in TCB at 120°C, in the presence of 2.2 eq. CuCl_2_, 2.2 eq. TPMA, and blue light irradiation. d,e,f) ^1^H NMR spectra of depolymerization kinetics under thermal, ICAR, and photothermal conditions.

Given that both higher CuCl_2_ concentration and reduced temperatures independently enhance the control of the system, we hypothesized that their combination could prove optimal. Under these dual conditions, the ATRP equilibrium is shifted decisively toward the dormant species alongside a suppressed depropagation rate, facilitating the frequent reversible termination required for the uniform unzipping of polymer chains. Indeed, for depolymerization of the homopolymer under these conditions (2.2 eq. CuCl_2_, 13 eq. TPMA with respect to end group, 130°C), experimental results closely mirrored the theoretical ideal, showing a strong correlation between *M*_n_ reduction and depolymerization conversion. Specifically, at 14% conversion, a 14% *M*_n_ shift was observed, in excellent agreement with the theoretical value of 14%. As the depolymerization proceeded, the molecular weight decreased almost proportionally to the conversion, with subsequent shifts of 28% and 39% at 32% and 53% conversion, respectively (Figure S13, S14 Table S9). The slight divergence from the ideal linear relationship at higher conversions (39% *M*_n_ shift instead of 53% at 53% conversion) was attributed to the presence of a small fraction non‐living chains originating from the synthesis of the polymer. The enhanced control of the system was further corroborated through the depolymerization of the diblock copolymer under identical conditions, which revealed a distinct sequential release of monomers. The MMA block remained undetectable via ^1^H NMR spectroscopy in the early stages of the reaction. Even at higher depolymerization conversions (55%), when BzMA had almost fully been released, only 22% of the total monomer detected was MMA, indicating high fidelity in the sequential unzipping of the blocks. Beyond this point, the rate of PBzMA recovery plateaued while MMA continued to be released until the final conversion was reached (Figure 3a,d, Table S10). Naturally, perfectly sequential monomer release remains unachievable due to the intrinsic dispersity of the polymers, and the unavoidable presence of a small amount of non‐living or defective chains. Taken altogether, these results demonstrate a robust controlled system that facilitates effective molecular weight control throughout the depolymerization process.

Finally, we evaluated the robustness of our methodology across different ATRP depolymerizations to establish its broader applicability. We found that the level of molecular weight control is independent of the catalyst activation pathway employed. Whether the deactivator was reduced via an excess of ligand, a radical initiator (ICAR), or blue light mediation, a similar level of control was consistently achieved (Figure 3a,b,c, Tables S10–S14). These results demonstrate that while high catalyst concentrations and regulated temperatures are fundamental requirements for control, the specific mode of initiation remains highly flexible. It is noted that although thermal ATRP depolymerization, photothermal ATRP depolymerization, and ICAR ATRP depolymerization resulted in comparable levels of control, ICAR depolymerization featured overall lower final conversion (66% for ICAR, 78% for photothermal and 82% for the thermal system) (Tables S13 and S14). The versatility of the system also extends to the coordination environment; substituting TPMA with common ligands such as PMDETA or Me_6_Tren yielded similar levels of precision (Figure S15, Tables S10, S15, S16). Collectively, these findings establish a highly adaptable ATRP depolymerization platform that can be tailored to various experimental constraints without compromising the control over the unzipping process.

## 3 Conclusions

In summary, we have established the first controlled ATRP depolymerization of polymethacrylates, overcoming the long‐standing challenge of unregulated chain unzipping. By strategically increasing catalyst concentrations and modulating reaction temperatures, we successfully tuned the ATRP equilibrium to improve deactivation with respect to depropagation. This kinetic regulation facilitates a linear reduction in molecular weight with conversion, representing the first true reversal of the ATRP mechanism. The precision of this methodology was demonstrated through selective, block‐by‐block depolymerization of copolymers, as well as its robust performance across thermal, photochemical, and ICAR activation platforms. By bridging precision polymer synthesis with controlled depolymerization, this approach expands the ATRP toolbox to exert control over both the creation and deconstruction of macromolecular architectures. Ultimately, this unlocks new capabilities for ATRP‐derived materials, ranging from on‐demand tuning of molecular weight‐dependent properties to high‐resolution structural characterization of copolymer sequences.

## 4 Experimental Section/Methods

### 4.1 Synthesis of Chlorine‐Terminated Poly(Benzyl Methacrylate) (PBzMA‐Cl) Homopolymer via ARGET ATRP

In a 5 mL glass vial 17.9 mg of CuCl_2_ and 83.41 µL *N*,*N*,*N*′,*N*′′,*N*′′‐pentamethyldiethylenetriamine (PMDETA) were dissolved in 2.7 mL acetonitrile, resulting in a blue solution. The solution was briefly sonicated in a benchtop sonication bath to aid the solubilization of the components. In a 100 mL round bottom flask equipped with a magnetic stir bar and charged with 20 mL benzyl methacrylate (118 mmol, 120 equiv.) and 8.0 mL anisole, 1 mL of the CuCl_2_/PMDETA stock solution (0.049 mmol, 0.05 equiv. CuCl_2_, 0.148 mmol, 0.15 equiv. PMDETA) was added, along with 169 µL of ethyl α‐chlorophenyl acetate (0.98 mmol, 1 equiv.). The flask was sealed with a rubber septum, and the polymerization mixture was deoxygenated via nitrogen purging for 20 min. Concurrently, a solution of tin(II) 2‐ethylhexanoate (Sn(EH)_2_) in anisole was prepared in a glass vial, sealed with a rubber septum, and deoxygenated for 20 min via nitrogen sparging. After deoxygenation, 0.5 mL (0.197 mmol, 0.2 equiv.) of the tin solution was transferred to a round‐bottom flask with a deoxygenated syringe, resulting in a deep blue solution. The flask was immediately placed into a 70°C oil bath with magnetic stirring (400 rpm). Samples for ^1^H NMR and SEC analysis were taken periodically under nitrogen. After 46 min, the polymerization was stopped at 42% polymerization conversion by removing the reaction from the oil bath and removing the septum, exposing the solution to air.

### 4.2 Purification of PBzMA‐Cl Homopolymer

The polymerization mixture was diluted with acetone and passed through a basic alumina column to remove the catalyst. The solution was then concentrated, precipitated in cold methanol, and filtered under vacuum. This process was repeated 3 times until all trace monomer had been removed. The purity of the polymer was confirmed via ^1^H NMR analysis. The purified polymer was dried in the vacuum oven for 24 h and analyzed via SEC to measure molar mass and dispersity.

### 4.3 Synthesis of Chlorine‐Terminated Poly(Methyl Methacrylate) (PMMA‐Cl) Macroinitiator via ARGET ATRP

In a 5 mL glass vial, 34.9 mg of CuCl_2_ and 81.3 µL of PMDETA were dissolved in 1.67 mL acetonitrile, resulting in a blue solution. The solution was briefly sonicated in a benchtop sonication bath to aid the solubilization of the components. In a 100 mL round bottom flask equipped with a magnetic stir bar and charged with 10 mL methyl methacrylate (93.5 mmol, 60 equiv.) and 4.0 mL anisole, 1 mL of the CuCl_2_/PMDETA stock solution (0.156 mmol, 0.1 equiv. CuCl_2_, 0.23 mmol, 0.15 equiv. PMDETA) was added, along with 267 µL of ethyl α‐chlorophenyl acetate (1.56 mmol, 1 equiv.). The flask was sealed with a rubber septum, and the polymerization mixture was deoxygenated via nitrogen purging for 20 min. Concurrently, a 311 mM solution of tin(II) 2‐ethylhexanoate (Sn(EH)_2_) in anisole was prepared in a glass vial, sealed with a rubber septum, and deoxygenated for 20 min via nitrogen sparging. After deoxygenation, 0.5 mL of the tin solution was transferred to the round‐bottom flask with a deoxygenated syringe, resulting in a deep blue solution. The flask was immediately placed into a 70°C oil bath with magnetic stirring. Samples for ^1^H NMR and SEC analysis were taken periodically under nitrogen. The polymerization was stopped at 51% polymerization conversion by removing the reaction from the oil bath and removing the septum, exposing the solution to air. The polymerization mixture was diluted with acetone and passed through a basic alumina column to remove the catalyst. The solution was then concentrated, precipitated in cold methanol, and filtered under vacuum. This process was repeated until all trace monomer had been removed. The purity of the polymer was confirmed via ^1^H NMR analysis. The purified polymer was dried in the vacuum oven for 24 h and analyzed via SEC to measure molar mass and dispersity.

### 4.4 Synthesis of Chlorine‐Terminated Poly(Methyl Methacrylate)–*b*–Poly(Benzyl Methacrylate) (PMMA‐*b*‐PBzMA‐Cl) Diblock Copolymer via ARGET ATRP

In a 50 mL round bottom flask, equipped with a stirring bar, 2.065 g of PMMA‐Cl macroinitiator (0.49 mmol, 1 equiv.) was dissolved in 2 mL of anisole, and 5 mL of BzMA (29.5 mmol, 60 equiv.) was added. A stock solution of CuCl_2_ and PMDETA was prepared in acetonitrile, out of which 1 mL (0.049 mmol, 0.1 equiv. CuCl_2_, 0.074 mmol, 0.15 equiv. PMDETA) was transferred to the flask, resulting in a pale blue solution. The reaction flask was sealed with a rubber septum and deoxygenated under nitrogen sparging for 20 min. Concurrently, a 196.7 mM solution of Sn(EH)_2_ in anisole was prepared, out of which 0.5 mL (0.098 mmol, 0.2 equiv.), was added to the reaction mixture with a deoxygenated syringe, resulting in the color of the solution turning deep blue. Immediately, the flask was submerged in a 70°C oil bath with magnetic stirring. The reaction was stopped at 56% conversion by removing it from the oil bath and opening the flask to air. The polymerization mixture was diluted with acetone and passed through a column filled with basic alumina to remove the metal catalyst. The diluted solution was then concentrated, precipitated in cold methanol, and filtered under vacuum. The process was repeated until no residual monomers were detected with ^1^H NMR analysis. The purified block copolymer was dried in the vacuum oven for 24 h.

### 4.5 Chain Extension of PBzMA‐Cl via ARGET ATRP for Livingness Determination

In a 10 mL test tube, equipped with a stirring bar, 78.8 mg of PBzMA‐Cl macroinitiator (0.00885 mmol, 1 equiv.) was dissolved in 0.1 mL of anisole, and 0.3 mL of BzMA (1.77 mmol, 200 equiv.) was added. A stock solution of CuCl_2_ and PMDETA was prepared in acetonitrile, out of which 10 µL (0.000885 mmol CuCl_2_, 0.1 equiv. CuCl_2_, 0.00132 mmol PMDETA, 0.15 equiv. PMDETA) was transferred to the flask, resulting in a pale blue solution. The reaction flask was sealed with a rubber septum and deoxygenated under nitrogen sparging for 20 min. Concurrently, a solution of Sn(EH)_2_ in anisole was prepared and deoxygenated, out of which 0.1 mL (0.00177 mmol, 0.2 equiv.), was added to the reaction mixture with a deoxygenated syringe. Immediately, the flask was submerged in a 70°C oil bath with magnetic stirring. Samples for SEC analysis were taken periodically in situ under a nitrogen blanket.

### 4.6 Example of Thermal Depolymerization Procedure for PBzMA‐Cl with CuCl_2_/ TPMA

Depolymerization is carried out in 1,2,4‐trichlorobenzene (TCB) using 0.22 equivalents CuCl_2_ and 1.3 equivalents TPMA. In a 10 mL test tube, 27.0 mg of PBzMA was added and dissolved in 3 mL of TCB (50 mM BzMA repeat unit concentration, RU). A stock solution of CuCl_2_ and TPMA was prepared by charging 3.0 mg CuCl_2_, 38.1 mg TPMA, and 0.34 mL DMF in a 5 mL vial. The solution was sonicated briefly to ensure full solubilization of the components. 10 µL of the stock solution (0.0007mmol CuCl_2,_ 0.22 eq. CuCl_2_, 0.0039 mmol TPMA, 1.3 eq. TPMA with respect to end group) was transferred to the reaction tube. The test tube was sealed with a rubber septum and deoxygenated via nitrogen purging for 15 min. Subsequently, it was submerged in a 170°C oil bath to start the depolymerization reaction. Samples for ^1^H NMR and SEC analysis were taken periodically in situ under a nitrogen blanket. For the depolymerization of PMMA‐*b*‐PBzMA‐Cl, identical conditions were used, and the reactant quantities were adjusted accordingly.

This is a representative example for the depolymerization procedure and was carried out similarly at 190°C, 130°C, 120°C under otherwise identical conditions.

### 4.7 Example of Thermal Depolymerization Procedure for PMMA‐*b*‐PBzMA‐Cl with CuCl_2_/ PMDETA

Depolymerization is carried out in 1,2,4‐trichlorobenzene (TCB) using 2.2 equivalents CuCl_2_ and 13 equivalents PMDETA with respect to end group. In a 10 mL test tube, 26.9 mg of PMMA‐*b*‐PBzMA were added and dissolved in 3 mL of TCB ([RU]_0_ = 50 mΜ). A stock solution of CuCl_2_ and PMDETA was prepared by charging 5 mg CuCl_2_, 46 µL PMDETA, and 147 µL DMF in a 5 mL vial. The solution was sonicated briefly to ensure full solubilization of the components. 20 µL of the stock solution (0.0051 mmol CuCl_2,_ 2.2 eq. CuCl_2_, 0.03 mmol PMDETA, 13 eq. PMDETA with respect to end group) was transferred to the reaction tube. The test tube was sealed with a rubber septum and deoxygenated via nitrogen purging for 15 min. Subsequently, it was submerged in a 130°C oil bath to start the depolymerization reaction. Samples for ^1^H NMR and SEC analysis were taken periodically in situ under a nitrogen blanket.

### 4.8 Example of Thermal Depolymerization Procedure for PBzMA‐Cl and (PMMA‐b‐PBzMA‐Cl) with CuCl_2_/ Me_6_Tren

Depolymerization is carried out in 1,2,4‐trichlorobenzene (TCB) using 2.2 equivalents CuCl_2_ and 13 equivalents Me_6_Tren. In a 10 mL test tube, 26.9 mg of PBzMA was added and dissolved in 3 mL of TCB ([RU]_0_ = 50 mΜ). A stock solution of CuCl_2_ and *Me_6_* Tren was prepared by charging 8.5 mg CuCl_2_, 100 µL Me_6_Tren, and 250 µL DMF in a 5 mL vial. The solution was sonicated briefly to ensure full solubilization of the components. 20 µL of the stock solution (0.0051 mmol CuCl_2,_ 2.2 eq. CuCl_2_, 0.03 mmol Me_6_Tren, 13 eq. Me_6_Tren with respect to end group) was transferred to the reaction tube. The test tube was sealed with a rubber septum and deoxygenated via nitrogen purging for 15 min. Subsequently, it was submerged in a 130°C oil bath to start the depolymerization reaction. Samples for ^1^H NMR and SEC analysis were taken periodically in situ under a nitrogen blanket.

### 4.9 Example of ICAR Depolymerization Procedure for (PMMA‐*b*‐PBzMA‐Cl) with CuCl_2_/ TPMA

Depolymerization is carried out in 1,2,4‐trichlorobenzene (TCB) using 2.2 equivalents CuCl_2_, 2.2 equivalents TPMA and 2.2 equiv. 1,1’‐Azobis(cyclohexanecarbonitrile) (ABCN) with respect to the end group. In a 10 mL test tube, 26.9 mg of PMMA‐*b*‐PBzMA was added and dissolved in 2.9 mL of TCB ([RU]_0_ = 50 mΜ). A stock solution of CuCl_2_ and TPMA was prepared by charging 5.5 mg CuCl_2_, 11.9 mg TPMA, and 161.8 µL DMF in a 5 mL vial. The solution was sonicated briefly to ensure full solubilization of the components. 20 µL of the stock solution (0.0051 mmol CuCl_2,_ 2.2 eq. CuCl_2_, 0.0051 mmol TPMA, 2.2 eq. TPMA with respect to end group) was transferred to the reaction tube. Concurrently, a stock solution of ABCN in TCB was prepared by charging 10 mg of ABCN and 0.8 mL TCB in a 5 mL vial. 0.1 mL of the stock solution was transferred to the reaction tube. The test tube was sealed with a rubber septum and deoxygenated via nitrogen purging for 15 min. Subsequently, it was submerged in a 120°C oil bath to start the depolymerization reaction. Samples for ^1^H NMR and SEC analysis were taken periodically in situ under a nitrogen blanket.

### 4.10 Example of Photothermal Depolymerization Procedure for (PMMA‐*b*‐PBzMA‐Cl) with CuCl_2_/ TPMA

Depolymerization is carried out in 1,2,4‐trichlorobenzene (TCB) using 2.2 equivalents CuCl_2_ and 2.2 equivalents TPMA. with respect to end group. In a 10 mL test tube, 26.9 mg of PMMA‐*b*‐PBzMA was added and dissolved in 2.9 mL of TCB ([RU]_0_ = 50 mΜ). A stock solution of CuCl_2_ and TPMA was prepared by charging 12.5 mg CuCl_2_, 27 mg TPMA and 367.6 µL DMF in a 5 mL vial. The solution was sonicated briefly to ensure full solubilization of the components. 20 µL of the stock solution (0.0051 mmol CuCl_2,_ 2.2 eq. CuCl_2_, 0.0051 mmol TPMA, 2.2 eq. TPMA with respect to end group) was transferred to the reaction tube. The test tube was sealed with a rubber septum and deoxygenated via nitrogen purging for 15 min. Subsequently, it was submerged in a 120°C oil bath to start the depolymerization reaction.

The solution was irradiated with 460 nm light (20.12 ± 1.10 mW/cm^2^). The reaction setup involved a 400 mL beaker that was surrounded 360° by an LED strip. The LED strip was purchased from Mxellex. Samples for ^1^H NMR and SEC analysis were taken periodically in situ under a nitrogen blanket.

### 4.11 Determination of the Relative Fraction of Regenerated Monomers During the Depolymerization of Block Copolymers

NMR samples that were prepared by direct sampling into deuterated chloroform were analyzed. The relative fraction of regenerated monomers (MMA/BzMA) during the depolymerization of PMMA‐*b*‐PBzMA) was calculated by comparing the intensities of the vinyl proton peaks in the 6–6.3 ppm region. Methyl Methacrylate monomer evaporates over time under the typical reaction conditions (130°C‐170°C). The MMA evaporation was accounted for by using the BzMA methylene peaks as an internal standard. More specifically, the total number of protons adjacent to the ester (attributed to PMMA, MMA, PBzMA and BzMA) should remain constant before and after depolymerization. By comparing the intensities of the −OCH_2_ BzMA and PBzMA peaks to the intensities of the −OCH_3_ MMA and PMMA peaks, the MMA evaporation percentage can be determined and incorporated into the analysis.

## Author Contributions

**Dimitra Mantzara**: investigation, Writing – original draft, data curation, visualization, methodology. **Richard Whitfield**: writing – review and editing, supervision, visualization, methodology. **Glen R. Jones**: writing – original draft, supervision, visualization, writing – review and editing, methodology. **Nghia P. Truong**: writing – review and editing, visualization, methodology. **Athina Anastasaki**: conceptualization, writing – review and editing, writing – original draft, supervision, resources, project administration, visualization, funding acquisition, methodology.

## Funding

ETH Zurich

## Conflicts of Interest

The authors declare no conflicts of interest.

## Supporting information




**Supporting File**: advs77962‐sup‐0001‐SuppMat.docx.

## Data Availability

Experimental procedures, characterization data, and all other relevant data supporting the findings of this study are available within the article and its supplementary information files.
