# Supplementary material for: Controlled Atom Transfer Radical Depolymerization
Source: Adv Sci (Weinh). 2026 Sep 27:e77962. Online ahead of print. doi: 10.1002/advs.77962 (PMC13616323; doi:10.1002/advs.77962)
Supplement: Supplementary file 1 [file ADVS-9999-e77962-s001.docx]

Supporting Information

Controlled Atom Transfer Radical Depolymerization

Dimitra Mantzara, Richard Whitfield, Glen R. Jones, Nghia P. Truong, and Athina Anastasaki*

**Materials:**

Benzyl methacrylate (>98.0%) was purchased from Tokyo Chemical Industry, and methyl methacrylate (99.0%) was purchased from Sigma-Aldrich. Both monomers were purified by filtration through basic alumina before use. Tris(2-pyridylmethyl)amine (97%) was purchased from AmBeed and was used as received. Tris-(2-(dimethylamino)ethyl)amine (Me_6_Tren) was synthesized according to literature procedure.^1^ The remaining materials were purchased either from Sigma-Aldrich or Fischer Scientific and were used as received.

**Instrumentation:**

**Proton nuclear magnetic resonance spectroscopy:**

^1^H NMR spectra were measured on a 300 MHz Bruker Avance spectrometer and deuterated chloroform (CDCl_3_) was used as the solvent. Chemical shifts are given in ppm downfield from tetramethylsilane and referenced to residual proton signals of the deuterated solvent. The depolymerization conversions were calculated by comparing the integrals of the monomeric vinyl proton (5.6 – 6.2 ppm) peaks to the integrals of the combined monomer and polymer methylene peaks (4.9 – 5.2 ppm).

**Size exclusion chromatography (SEC)**

Size exclusion chromatography (SEC) was measured on a modular Shimadzu instrument, comprising a CBM-20A system controller, a LC-20AD pump, an SIL-20A automatic injector and a 10.0 μm bead size guard column (50 × 7.5 mm) connected to three KF-805L columns (300 × 8 mm, 10 μm bead size, 5000 Å maximum pore size), an SPD-20A ultraviolet detector, and an RID-20A differential refractive index detector. The temperature of the columns was maintained at 40°C, by a CTO-20A oven. *N,N*-dimethylacetamide (HPLC grade, containing 0.03% w/v LiBr) was used as the mobile phase, with a flow rate of 1 mL/min. The molecular weight calibration was performed using poly(methyl methacrylate) standards, with molecular weights ranging from 5000 to 1.5 × 10⁶ Da (Agilent Technologies). All SEC samples were dissolved in dimethylacetamide, filtered through basic alumina to remove any metal catalyst residuals, and finally passed through a 0.45 μm PTFE filter before analysis.

**d**

**H_2_O**

**c**

**b**

**a**

Figure S1: ^1^H NMR spectrum of the purified PBzMA-Cl homopolymer.

Figure S2 : SEC trace of the purified PBzMA-Cl homopolymer.

Figure S3: Deconvoluted SEC trace of the chain-extended PBzMA-Cl polymer to quantify livingness. Livingness was quantified by comparing the integration area of the unreacted PBzMA-Cl polymer to the area of the chain extended PBzMA-*b*-PBzMA-Cl polymer based on the methodology reported by Wooley and coworkers.^2^

**a,a’**

**b,b’**

**c’**

**c**

**d**

Figure S4: ^1^H NMR spectrum of the purified PMMA-*b*-PBzMA-Cl block copolymer.

Table S1: Polymerization data for the synthesis of the PMMA-*b*-PBzMA-Cl block copolymer.

| **Polymer** | **[M]/ECPA** | **Conv.** | ***M*_n_ ^(theor.)^** | ***M*_n_ ^(SEC)^** | ***Đ*** | **mol % BzMA (NMR)** | **Total DP** |
| --- | --- | --- | --- | --- | --- | --- | --- |
| **PMMA-Cl** | 60/1 | 52 % | 3,300 | 4,200 | 1.16 | 0 | 31 |
| **PMMA-*b*-PBzMA-Cl** | 60/1 | 56% | 9,200 | 9,300 | 1.11 | 52 | 65 |

Figure S5: SEC traces of the 1^st^ PMMA block (green) and the purified PMMA-*b*-PBzMA-Cl block copolymer (blue).

Table S2: Depolymerization of PBzMA-Cl in TCB using 0.22 eq. CuCl_2_ and 1.3 eq. TPMA with respect to end group ([RU]_0_ = 50 mM, T = 170 °C).

| **Depol. %** | ***M*_n_^(SEC)^** | ***Đ*** |
| --- | --- | --- |
| 0 | 8,800 | 1.17 |
| 13 | 8,400 | 1.20 |
| 20 | 8,000 | 1.24 |
| 38 | 7,500 | 1.26 |
| 60 | 7,100 | 1.27 |
| 84 | 6,900 | 1.28 |

Figure S6: SEC traces during the depolymerization of PBzMA-Cl in TCB using 0.22eq. CuCl_2_ and 1.3 eq TPMA with respect to end group ([RU]_0_ = 50 mM, T = 170 °C).

Figure S7: Depolymerization of PMMA-*b*-PBzMA-Cl in TCB using 0.22 eq. CuCl_2_ and 1.3 eq. TPMA with respect to end group ([RU]_0_ = 50 mM, T = 170 °C).

Table S3: Uncontrolled depolymerization of PBzMA-Cl in TCB using 0.022 eq. CuCl_2_ and 0.13 eq. TPMA with respect to end group ([RU]_0_ = 50 mM, T = 170 °C).

| **Depol. %** | ***M*_n_^(SEC)^** | ***Đ*** |
| --- | --- | --- |
| 0 | 8,700 | 1.19 |
| 30 | 8,400 | 1.22 |
| 67 | 8,100 | 1.23 |

Figure S8: Uncontrolled depolymerization of PMMA-*b*-PBzMA-Cl in TCB using 0.022 eq. CuCl_2_ and 0.13 eq. TPMA with respect to end group ([RU]_0_ = 50 mM, T = 170 °C).

Table S4: Partially controlled depolymerization of PBzMA-Cl in TCB using 2.2 eq. CuCl_2_ and 13 eq. TPMA with respect to end group ([RU]_0_ = 50 mM, T = 170 °C).

| **Depol. %** | ***M*_n_^(SEC)^** | ***Đ*** |
| --- | --- | --- |
| 0 | 8,600 | 1.19 |
| 26 | 7,200 | 1.26 |
| 71 | 5,400 | 1.28 |

Figure S9: Partially controlled depolymerization of PMMA-*b*-PBzMA-Cl in TCB using 2.2 eq. CuCl_2_ and 13 eq. TPMA with respect to end group ([RU]_0_ = 50 mM, T = 170 °C).

Table S5: Polymerization of BzMA varying the temperature under identical ARGET ATRP conditions. The polymerization conditions were: BzMA:ECPA:CuCl_2_:PMDETA:Sn(EH)_2_ = 120:1:0.05:0.15:0.2, Solvent= Anisole, [BzMA] _0_ = 3 M.

| **Temperature, °C** | **Time, min** | **Conv.** | ***M*_n_ ^(theor.)^** | ***M*_n_ ^(SEC)^** | ***Đ*** |
| --- | --- | --- | --- | --- | --- |
| **70** | 150 | 51% | 11,000 | 8,600 | 1.16 |
| **100** | 30 | 52% | 11,200 | 9,000 | 1.22 |
| **120** | 15 | 51% | 11,000 | 8,600 | 1.25 |
| **140** | 10 | 48% | 10,300 | 8,100 | 1.30 |
| **170** | 7 | 52 % | 10,800 | 8,400 | 1.35 |

Figure S10: SEC traces of polymers obtained by ARGET ATRP under varying temperatures.

Table S6: Depolymerization of PBzMA-Cl in TCB using 0.22 eq. CuCl_2_ and 1.3 eq. TPMA with respect to end group. ([RU]_0_ = 50 mM, T = 190 °C).

| **Depol. %** | ***M*_n_^(SEC)^** | ***Đ*** |
| --- | --- | --- |
| 0 | 8,900 | 1.19 |
| 28 | 8,300 | 1.20 |
| 44 | 7,700 | 1.25 |
| 52 | 7,600 | 1.26 |
| 60 | 7,300 | 1.29 |
| 70 | 7,300 | 1.28 |

Table S7: Depolymerization of PBzMA-Cl in TCB using 0.22 eq. CuCl_2_ and 1.3 eq. TPMA with respect to end group ([RU]_0_ = 50 mM, T = 130 °C).

| **Depol. %** | ***M*_n_^(SEC)^** | ***Đ*** |
| --- | --- | --- |
| 0 | 8,900 | 1.19 |
| 23 | 7,300 | 1.24 |
| 41 | 6,700 | 1.29 |
| 51 | 6,200 | 1.31 |
| 67 | 5,700 | 1.37 |

Figure S11: Depolymerization of PBzMA-Cl in TCB using 0.22 eq. CuCl_2_ and 1.3 eq. TPMA with respect to end group ([RU]_0_ = 50 mM, T = 120 °C).

Figure S12: Depolymerization of PMMA-b-PBzMA-Cl in TCB using 0.22 eq. CuCl2 and 1.3 eq. TPMA with respect to end group ([RU]0 = 50 mM, T = 190 °C)

Table S8: Depolymerization of PMMA-*b*-PBzMA-Cl in TCB using 0.22 eq. CuCl_2_ and 1.3 eq. TPMA with respect to end group ([RU]_0_ = 50 mM, T = 130 °C).

| **Time (mins)** | **Depol. %** | **% BzMA** | **% MMA** |
| --- | --- | --- | --- |
| 15 | 2 | 100 | 0 |
| 30 | 8 | 95 | 5 |
| 45 | 16 | 89 | 11 |
| 60 | 25 | 82 | 18 |
| 120 | 49 | 76 | 24 |
|  |  |  |  |

Table S9: Controlled depolymerization of PBzMA-Cl in TCB using 2.2 eq. CuCl_2_ and 13 eq. TPMA with respect to end group ([RU]_0_ = 50 mM, T = 130 °C).

| **Depol. %** | ***M*_n_^(SEC)^** | ***Đ*** |
| --- | --- | --- |
| 0 | 8,700 | 1.19 |
| 14 | 7,400 | 1.20 |
| 25 | 6,900 | 1.26 |
| 32 | 6,300 | 1.28 |
| 53 | 5,300 | 1.37 |
| 82 | 6,300 | 1.45 |
|  |  |  |

Figure S13: SEC traces of controlled depolymerization of PBzMA-Cl in TCB using 2.2 eq. CuCl_2_ and 13 eq. TPMA with respect to end group ([RU]_0_ = 50 mM, T = 130 °C).

Figure S14: Comparison of the M_n_ shift evolution for the uncontrolled ATRP system (0.022 eq. CuCl_2_, 0.13 eq. TPMA, 170°C), the ‘traditional’ ATRP depolymerization system (0.22 eq. CuCl_2_, 1.3 eq. TPMA, 170°C) and the controlled thermal ATRP depolymerization system (2.2 eq. CuCl_2_, 13 eq. TPMA, 130°C), plotted against the theoretical M_n_ shift values that should be observed during controlled depolymerization.

Table S10: Controlled depolymerization of PMMA-*b*-PBzMA-Cl in TCB using 2.2 eq. CuCl_2_ and 13 eq. TPMA with respect to end group ([RU]_0_ = 50 mM, T = 130 °C).

| **Time (mins)** | **Depol. %** | **% BzMA** | **% MMA** |
| --- | --- | --- | --- |
| 20 | 10 | 100 | 0 |
| 22 | 15 | 100 | 0 |
| 24 | 22 | 98 | 2 |
| 25 | 26 | 96 | 4 |
| 26 | 31 | 94 | 6 |
| 27 | 34 | 91 | 9 |
| 28 | 40 | 89 | 11 |
| 29 | 43 | 85 | 15 |
| 30 | 47 | 84 | 16 |
| 32 | 55 | 78 | 22 |
| 35 | 63 | 70 | 30 |
| 45 | 79 | 56 | 44 |

Table S11: Controlled ICAR depolymerization of PMMA-*b*-PBzMA-Cl in TCB using 2.2 eq. CuCl_2_, 2.2 eq. TPMA and 2.2 eq ABCN with respect to end group. ([RU]_0_ = 50 mM, T = 120 °C).

| **Time (mins)** | **Depol. %** | **% BzMA** | **% MMA** |
| --- | --- | --- | --- |
| 10 | 1 | 100 | 0 |
| 12 | 10 | 100 | 0 |
| 15 | 29 | 95 | 5 |
| 20 | 47 | 82 | 18 |
| 25 | 57 | 75 | 25 |
| 30 | 62 | 69 | 31 |
| 45 | 68 | 63 | 37 |

Table S12: Controlled photothermal depolymerization of PMMA-*b*-PBzMA-Cl in TCB using 2.2 eq. CuCl_2_, 2.2 eq. TPMA with respect to end group. The reaction was performed under blue light irradiation ([RU]_0_ = 50 mM, T = 120 °C).

| **Time (mins)** | **Depol. %** | **% BzMA** | **% MMA** |
| --- | --- | --- | --- |
| 60 | 9 | 100 | 0 |
| 75 | 16 | 100 | 0 |
| 90 | 25 | 99 | 1 |
| 120 | 39 | 91 | 9 |
| 150 | 62 | 70 | 30 |

Table S13: Controlled ICAR depolymerization of PBzMA-Cl in TCB using 2.2 eq. CuCl_2_, 2.2 eq. TPMA and 2.2 eq ABCN with respect to end group. ([RU]_0_ = 50 mM, T = 120 °C).

| **Depol. %** | ***M*_n_^(SEC)^** | ***Đ*** |
| --- | --- | --- |
| 0 | 9,600 | 1.16 |
| 13 | 8,500 | 1.21 |
| 26 | 7,700 | 1.24 |
| 45 | 6,700 | 1.31 |
| 53 | 6,300 | 1.34 |
| 66 | 6,500 | 1.37 |

Table S14: Controlled photothermal depolymerization of PMMA-*b*-PBzMA-Cl in TCB using 2.2 eq. CuCl_2_, 2.2 eq. TPMA with respect to end group. The reaction was performed under blue light irradiation ([RU]_0_ = 50 mM, T = 120 °C).

| **Depol. %** | ***M*_n_^(SEC)^** | ***Đ*** |
| --- | --- | --- |
| 0 | 9,600 | 1.16 |
| 14 | 8,400 | 1.21 |
| 45 | 6,600 | 1.24 |
| 65 | 6,000 | 1.31 |
| 78 | 6,400 | 1.42 |

Figure S15: Comparison of our optimized controlled depolymerization protocol of PBzMA-Cl using TPMA versus PMDETA versus Me_6_TREN. All reactions were performed in TCB using 2.2 eq. CuCl_2_ and 13 eq. of the ligand, with respect to end group ([RU]_0_ = 50 mM, T = 130 °C).

Figure S16: Comparison of traditional uncontrolled depolymerization of PBzMA-Cl using TPMA versus PMDETA versus Me_6_TREN. All reactions were performed in TCB using 0.22 eq. CuCl_2_ and 1.3 eq. of the ligand, with respect to end group ([RU]_0_ = 50 mM, T = 170 °C).

Table S15: Controlled depolymerization of PMMA-*b*-PBzMA-Cl in TCB using 2.2 eq. CuCl_2_ and 13 eq. PMDETA with respect to end group. ([RU]_0_ = 50 mM, T = 130 °C).

| **Time (mins)** | **Depol. %** | **% BzMA** | **% MMA** |
| --- | --- | --- | --- |
| 25 | 8 | 100 | 0 |
| 28 | 10 | 100 | 0 |
| 30 | 11 | 100 | 0 |
| 35 | 16 | 100 | 0 |
| 40 | 20 | 98 | 2 |
| 50 | 28 | 96 | 4 |
| 60 | 38 | 90 | 10 |
| 75 | 57 | 73 | 27 |
| 90 | 69 | 61 | 39 |
| 120 | 73 | 57 | 43 |

Table S16: Controlled depolymerization of PMMA-*b*-PBzMA-Cl in TCB using 2.2 eq. CuCl_2_ and 13 eq. Me_6_(TREN) with respect to end group ([RU]_0_ = 50 mM, T = 130 °C).

| **Time (mins)** | **Depol. %** | **% BzMA** | **% MMA** |
| --- | --- | --- | --- |
| 25 | 4 | 100 | 0 |
| 28 | 6 | 100 | 0 |
| 30 | 7 | 100 | 0 |
| 35 | 11 | 100 | 0 |
| 40 | 21 | 97 | 3 |
| 50 | 25 | 96 | 4 |
| 60 | 37 | 90 | 10 |
| 75 | 49 | 80 | 20 |
| 90 | 61 | 68 | 32 |
| 120 | 73 | 57 | 43 |

(1) Ciampolini, M.; Nardi, N. Five-Coordinated High-Spin Complexes of Bivalent Cobalt, Nickel, andCopper with Tris (2-dimethylaminoethyl) amine. *Inorganic chemistry* **1966**, *5* (1), 41-44.

(2) Bartels, J. W.; Cauët, S. I.; Billings, P. L.; Lin, L. Y.; Zhu, J.; Fidge, C.; Pochan, D. J.; Wooley, K. L. Evaluation of isoprene chain extension from PEO macromolecular chain transfer agents for the preparation of dual, invertible block copolymer nanoassemblies. *Macromolecules* **2010**, *43* (17), 7128-7138.
